# Developing physical seed priming as a practical tool for stress-resilient crop production

**DOI:** 10.1042/EBC20250053

**Published:** 2026-07-30

**Authors:** Eduardo A. Zelada Lau, Michael R. Roberts

**Affiliations:** Lancaster Environment Centre, Lancaster University, Lancaster LA1 4YQ, U.K.

**Keywords:** Abiotic stress, Germination, Physical stimulus, Seed priming

## Abstract

Seed priming is a common approach to improve germination and seedling stress tolerance. Most priming treatments involve imbibition of seeds in water or in solutions of different chemicals, followed by redrying seed before the completion of germination. Physical priming treatments based on various forms of radiation or non-thermal plasma, for example, are also possible. Physical priming has some advantages over imbibition-based priming treatments, including avoiding negative impacts on the longevity of primed seed. Physical priming has potential for applications in agriculture, but questions remain about mechanisms of action and the range of outcomes generated by different types of treatment. Here, we suggest potential mechanisms by which physical treatments might impact the future performance of seeds and seedlings, including physiochemical modifications of the seed coat, generation of reactive oxygen species, and DNA-damage responses. In particular, we discuss how biological responses may be triggered by treatment of dry seeds that lack significant metabolic activity. We also briefly consider downstream molecular and biochemical responses to physical priming in germinating seeds. We conclude our review by briefly reflecting on key steps required for effective commercial exploitation.

## Introduction

Climate change represents a serious threat to agricultural productivity [1,2]. This is largely a consequence of abiotic stress resulting from changes in temperature and water availability [2]. Climate change impacts are further exacerbated by anthropogenic pollution and soil degradation [3]. Seed germination represents a critical stage of agricultural production. Germination rate and synchronicity, alongside tolerance to abiotic stress, are key factors in crop establishment and productivity [4]. The production of high-quality seed is therefore an important tool in maximising agricultural and horticultural productivity, and the global seed industry was worth an estimated US$77 billion in 2025 [5].

**Figure 1:**
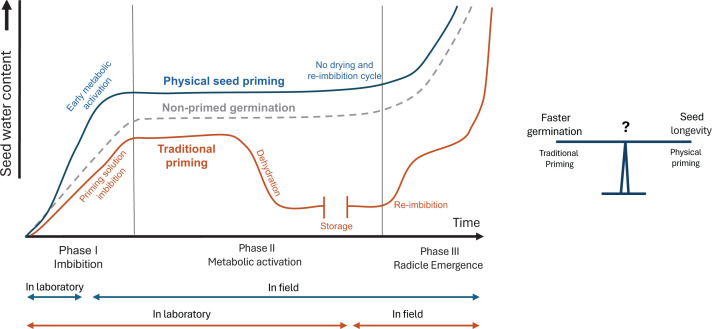
Comparison of the impacts of solute-based and physical priming on seed germination Seed water uptake relations during priming and germination of non-primed (grey dotted line), traditionally primed (orange line), and physically primed (blue line) seed. Upon imbibition, seed water uptake is characterised by three distinct phases [6]. During traditional priming, seeds are imbibed in a priming solution to trigger pre-germinative metabolic events (phase II) and dehydrated for storage before entering phase III. Subsequent re-imbibition enables accelerated germination. For physical priming, dry seed treatment eliminates dehydration/re-imbibition processes. Since early repair mechanisms and/or signaling processes occur, physical primed seeds may display early metabolic activation. This reduces the time spent in laboratory settings, enhancing its scalability. (Partially adapted from [12]). Finally, a trade-off is depicted where the key benefit from each priming method must be prioritised according to the farmer’s needs.

One common approach to improve seed vigour is priming [7,8]. In its simplest form, seed priming involves soaking seeds in water (hydropriming) followed by redrying before germination proceeds to radical emergence. Germination is characterised by two initial phases: an initial rapid water uptake (phase I) followed by the activation of energy metabolism, *de novo* transcription and protein translation, and protective responses including DNA and membrane repair (phase II) [6], events referred to as ‘pre-germinative metabolism’. During priming, seeds are hydrated up to the second phase of seed germination and are thus able to complete germination more rapidly and synchronously upon subsequent re-imbibition, since phase II processes are at least partially complete (Figure 1). This basic method can be modified by imbibing seeds in solutions containing osmotica, nutrients, phytohormones, etc., that have a range of impacts on germination and post-germination performance [7,8]. Importantly, yield benefits from simple on-farm priming systems have been confirmed [9].

Whilst these seed priming treatments have been widely successful, there are also drawbacks. Imbibition and redrying often reduces the subsequent longevity of seeds [10], and can result in pollution via transfer of priming chemicals to the field. Solute-based priming also poses problems in scalability. More recently, physical priming has emerged as a promising alternative [11–14]. Physical priming treatments include exposure of seeds to various forms of non-thermal plasma (NTP), electromagnetic fields, ultraviolet (UV) radiation, microwaves, and ionising radiation. Like traditional priming methods, physical treatments often accelerate germination, enhance seedling vigour, and in some cases, increase the defence capacity of primed plants against abiotic stress [12,14,15]. Physical methods have several advantages over traditional priming methods, including minimising pollution. Importantly, since physical priming is a non-hydration pre-sowing treatment, reduced longevity of primed seeds caused by DNA damage during imbibition and redrying can potentially be overcome.

Despite documented benefits of physical seed priming for stress tolerance and germination, we lack fundamental understanding of how dry seeds perceive and transduce physical signals into lasting physiological changes. Physical priming agents interact with seeds in fundamentally different ways to solute-mediated treatments and yet can generate similar outcomes. This gap in understanding currently limits the optimisation and wider application of physical priming. In the present review, we attempt to synthesise current evidence on sensing and signalling mechanisms and downstream responses, to build a mechanistic framework for physical priming.

## Physical seed priming to combat abiotic stress

Seed priming can contribute to climate change resilience at multiple levels. Firstly, it can improve germination performance in non-optimal conditions, such as under low water availability, high salinity, or high or low temperature. Secondly, there may also be impacts on seedling vigour that improve post-germination establishment. Finally, some priming treatments also establish long-term memory that can enhance responses to stress experienced later during the life of the plant. Physical priming methods vary widely in their nature, and the outcomes of a particular treatment will vary depending on factors such as wavelength, dose, and duration of exposure. Outcomes will also vary between species, depending on seed morphology and physiology. Table 1 lists a selection of recent examples of physical priming treatments that generate improvements in germination and/or stress tolerance, and the reader is referred to more comprehensive reviews of the area for more in-depth coverage of physical priming treatments [11–16].

**Table 1:** Recent examples of physical priming treatments that improve germination, plant growth, and/or stress tolerance

Priming agent	Plant species	Germination improved?	Plant growth improved?	Whole plant stress tolerance improved?	Reference
UV-B	Rice	nd	nd	Improved growth under salt stress. Various physiological parameters affected.	[17]
UV-B	Rice	nd	Yes—seedling stage, increase salinity stress	nd	[18]
UV-C	Lettuce	nd	nd	Mixed effects—some growth parameters improved by UV-C priming in the absence of stress, others in the presence of salt stress.	[19]
UV-C	Wheat	nd	nd	Improved resistance to biotrophic fungal pathogens	[20]
Gamma radiation	*Arabidopsis thaliana*	nd	No	Enhanced tolerance to mild salinity stress when imbibed seeds, but not dry seeds, irradiated	[21]
Gamma radiation	Quinoa	Yes	Yes (dose-dependent)	nd	[22]
Microwave radiation	Barley	Yes	Yes—seedling stage. Linked to changes in ROS and hormones.	nd	[23]
NTP	*Andrographis paniculata*	Yes	nd	nd	[24]
NTP	Norway spruce	Yes	Yes (dose-dependent). Adult stage: increase height and number of branches	nd	[25]
NTP	*Prosopis koelziana*	Yes, under control and salt stress	Yes—seedling stage, under control and salt stress.	Improved seedling growth under salt stress	[26]
NTP	Rapeseed	nd	Yes—adult stage under drought stress	Improved growth under drought stress. Various physiological and metabolic parameters affected.	[27]
NTP	Rice	Yes, under heat stress	nd	nd	[28]
NTP	Tomato	Yes	Yes—seedling stage	Improved plant growth under osmotic stress	[29]
NTP	Tomato	nd	nd	Cold stress tolerance improved via ROS and ABA signalling.	[30]
NTP	Tomato	Yes	Yes—seedling stage	Increase resistance to tomato brown rugose fruit virus	[31]
NTP	Tomato	Yes	Yes—seedling stage	Yes—biotic stress	[32]
NTP	Wheat	Yes	Yes—seedling stage	Improved seedling growth under drought stress	[33]
Ultrasound	Wheat	nd	Yes—seedling stage. Linked to DEGs and DMRs for Psyn, TCA cycle, and auxin.	nd	[34]

Abbreviation: nd; not determined.

## What are the mechanistic gaps in how seeds sense physical priming agents?

A major limitation in research in this area is the variability in treatment methodologies, test species, and growing conditions. Despite this heterogeneity, most research demonstrates that physical treatments optimise germination and stress tolerance parameters in a variety of seed crops and trees [11,12,15,16]. However, most reports to date focus on physiological outcomes rather than underlying mechanisms. Understanding the molecular pathways mediating physical priming poses a significant challenge yet is essential for developing optimised treatment protocols. Critically, it remains unclear how seeds perceive physical priming agents. One possible exception is UV-B radiation, where the well-characterised UV RESISTANCE LOCUS 8 (UVR8) photoreceptor has been proposed as a mediator of UV priming responses [12]. Whilst we can detect its expression across seed tissues in public transcriptome data, no study has demonstrated a functional role for UVR8 during germination, and its possible involvement in UV-mediated seed priming therefore remains to be proven. Regardless of the presence of receptor systems, a key gap in understanding comes from the fact that unlike traditional priming there is no solute uptake to activate regulatory pathways [35], or induction of pre-germinative metabolism through a controlled hydration–dehydration cycle [7]. Physical priming agents typically make first contact with the seed coat. Therefore, physicochemical modifications to the outer or inner layers of the dry seed may be one way in which physical treatments impact subsequent seed performance.

### Physical impacts on seed structure

**Figure 2:**
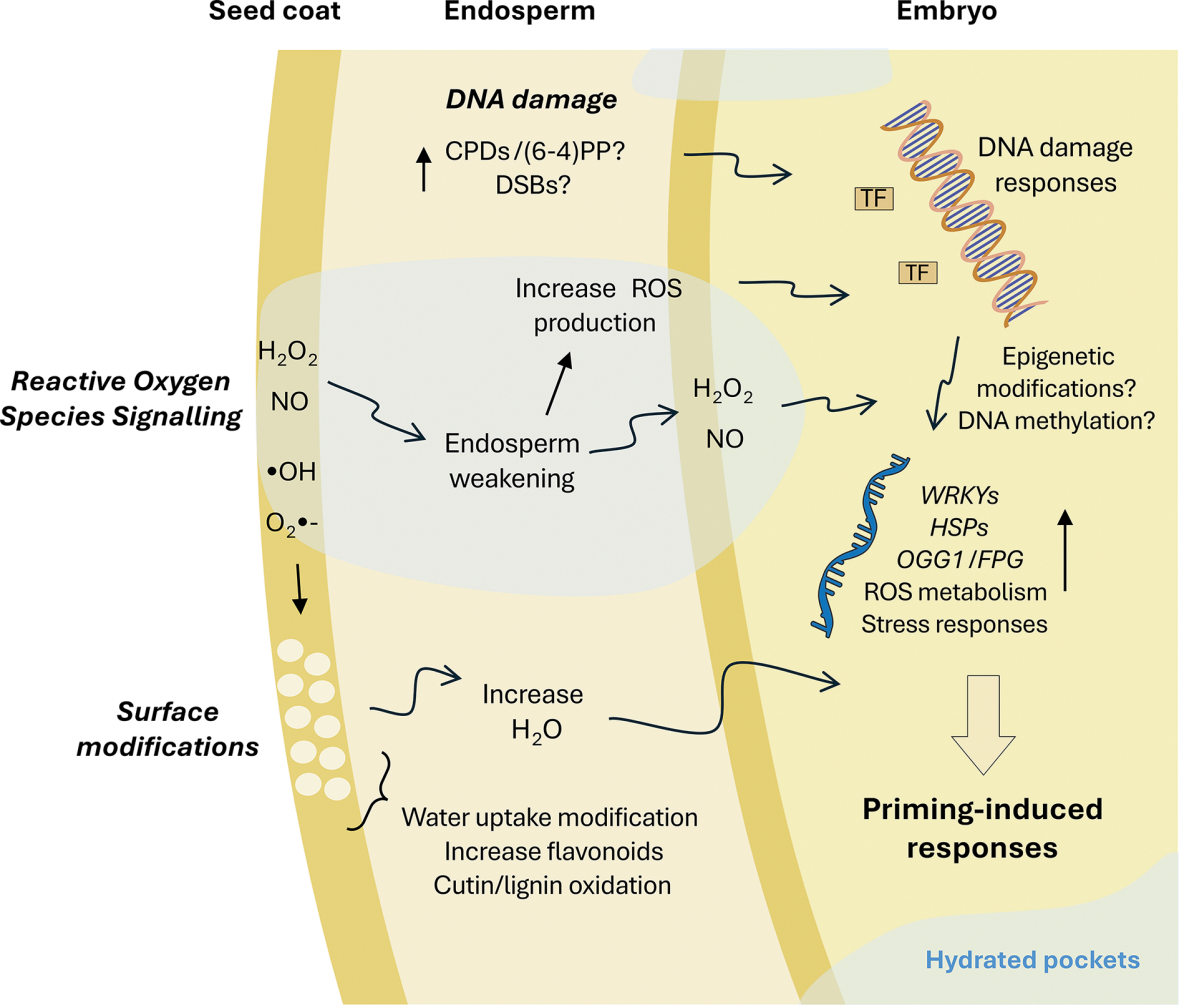
Integrated hypothetical model of signaling pathways involved in physical seed priming across seed tissues, synthesising current evidence and proposed mechanisms Upon physical priming treatment, surface modifications of the seed coat will modify water uptake, and increase flavonoid accumulation and cutin/lignin oxidation [29,36–40]. Similarly, short half-life ROS (•OH, O_2_•^−^) will also impact these modifications [14,15,43]. Additionally, longer half-life ROS (H_2_O_2_, NO) are likely to penetrate deeper seed layers, triggering endosperm weakening [18,44–48] and DNA-damage responses (DDRs) [46,47,53]. DNA damage induced during physical priming triggers DDRs upon imbibition [15,46,47]. It is unclear whether epigenetic modifications and/or DNA methylation marks mediate DDRs [28,34,87]. Widespread transcriptional modifications after priming could mediate the observed priming-induced responses [24,28,33,43,76,79,80,82–85]. Lines ending in arrows indicate positive interactions. H_2_O_2_, hydrogen peroxide; NO, nitric oxide; •OH, hydroxyl radicals; O_2_•^−^, superoxide; CPDs, cyclobutane pyrimidine dimers; (6-4)PPs, pyrimidine-pyrimidone (6-4) photoproducts; DSBs, double-strand breaks; WRKYs, WRKY transcription factor family; HSPs, heat shock proteins; OGG1, 8-oxoguanine DNA glycosylase; FPG, formamido-pyrimidine-DNA-glycosylase.

Unlike solutes that can reach the embryo and inner layers of the seed, it is likely that physical priming agents act primarily on the seed coat (testa) of dry seeds. Seed coat anatomy varies widely, but the testa typically comprises one or two layers of maternally-derived integument. The integuments are often toughened with lignin and contain a variety of other phenolics. They are usually covered by an outer cuticular layer and some also contain a mucilaginous epidermal layer. The testa is typically impermeable to water and/or oxygen and may contain chemical inhibitors of germination. A range of studies suggest that NTP and UV, in particular, often cause seed coat modifications. Microstructural or physiochemical modifications to the seed coat might mediate differences in germination parameters by altering permeability of the seed coat (Figure 2). If the restriction force imposed by the seed coat/endosperm or the seed capacity to regulate water uptake is altered, the overall relationship between the embryo and the surrounding tissues is modified, influencing radicle emergence [6]. Several studies support this idea. Recent research [29] observed that NTP treatment mechanically damaged the tomato seed coat, enhancing water uptake. Similarly, improved seed germination driven by modifications in the seed surface has been observed in quinoa [36] and pea [37]. Different components of the seed coat might have specific roles in terms of protecting seed structures and therefore in priming. Changes in the upper layers of the cuticle have been reported upon NTP treatment, possibly aiding water absorption [38]. Germination of seeds that lack polymers such as suberin and cutin is not improved by NTP, supporting their relevance in the priming treatment [39]. UV radiation can also alter seed coat properties. UV-C exposure during early imbibition induced the accumulation in bean seed coats of major flavonoids and *de novo* synthesis of soyasaponin, potentially mediating the observed germination improvements [40]. Despite these observations, there is much to learn about how deeply physical priming treatments penetrate the seed and the role of seed coat modifications in priming responses.

Heat has also been proposed as a possible component of some physical treatments, especially NTP and UV radiation. To date, few studies have tracked changes in temperature that seeds undergo during treatment, but Waskow et al. [14] suggested that heat flux generated during NTP exposure would have a limited contribution to any priming response. Findings suggest negligible effects from temperature, either because there is only a small increase in seed surface temperature (24–28°C), or because incubating seeds to a similar temperature is ineffective [15,41,42]. No reports have been made so far linking temperature changes during seed treatments with UV radiation to priming effects. Unlike NTP, UV treatments can span from minutes to several hours long, but seed surface temperatures exceeding 28–30°C seem unlikely considering reported UV exposure setups [12]. Nevertheless, this component should be assessed in further UV priming experiments.

### ROS as central signals for seed priming

Regardless of whether modifications to the seed coat contribute to the priming phenotype, multiple studies indicate that reactive oxygen species (ROS) and reactive nitrogen species (RNS) constitute early signalling molecules triggering biological responses following physical priming [14,15,43]. Upon imbibition, ROS function as signalling molecules along the embryonic axis for promoting germination. Some of the ROS/RNS produced upon NTP or UV exposure, including hydroxyl radicals (•OH) and superoxide (O_2_•^−^), are characterised by a short half-life [15]. These species could be involved in seed coat modifications, but it is unlikely they could act deeper than this. Other species such as hydrogen peroxide (H_2_O_2_) and nitrogen monoxide (NO) are likely to reach deeper layers given their longer half-lives and ability to cross membranes [15], and are known to participate in germination [44]. Physical constraints surrounding the embryo need to be relieved through cell wall weakening mechanisms. In imbibed seeds, ROS diffusion from the seed coat inwards promotes endosperm weakening, while endosperm changes may also influence outer layer properties [45]. It has been shown that NTP priming of *Arabidopsis* seed also triggers early ROS-mediated endosperm weakening [46]. ROS production upon physical priming has also been reported in wheat [47], rice [18], and *Arabidopsis* [48]. Therefore, ROS generation and signalling may have important roles mediating physical priming (Figure 2).

It is unclear how ROS/RNS signals generated in dry seeds are transduced to internal tissues. In dry seed, activity of ROS signalling pathways is minimal, being constrained by water availability. One possibility is that small hydrated pockets inside dry seeds [49] permit limited ROS accumulation and persistence after treatment despite minimal metabolic activity (Figure 2). Recently, August et al. [41] showed that *Arabidopsis* seeds with a 30% water content had a larger improvement in seed germination parameters after NTP treatment than those with 3% or 10% water. These results support the idea that accumulated ROS in these hydrated pockets can persist until full imbibition, triggering an amplified downstream response. Where this includes stimulation of antioxidant production, this could contribute to future seedling abiotic stress tolerance.

Other important questions are how different ROS arise, whether they have different roles, and the dynamics of ROS movement. While H_2_O_2_ diffuses passively across membranes, NO may enter cells through aquaporins [14]. Signals triggered in specific vegetative tissues can be propagated into other tissues [50], but whether and how ROS might traverse seed tissues is unknown. *In situ* localisation showed that during early imbibition, mitochondria are the primary intracellular ROS source [51], while later imbibition is associated with apoplastic ROS production [52]. Whether physical priming might also influence mitochondrial or apoplastic ROS production is unknown. H_2_O_2_ and NO signalling are closely linked with hormones and gene regulation processes related to germination [44], and both participate in NTP priming [43]. Nevertheless, their possible involvement as a regulatory hub during pre-germinative metabolism following physical seed priming needs to be elucidated.

### DNA-damage responses

Along with ROS and seed coat modifications, another likely core feature of priming is the DDR [53] (Figure 2). Preserving genome integrity in the seed is fundamental for successful seedling establishment and long-term seed stability [8,54,55]. DNA damage in seeds can be caused by exogenous factors such as UV, heat, or prolonged periods of unfavourable storage conditions, or endogenous factors such as ROS from metabolic by-products [56]. Seeds have therefore evolved spatio-temporally specialised DDR mechanisms to resolve genome lesions [57]. Pre-germination DDR mechanisms have been studied in detail, since they are critical to overall seed vigour and viability [58]. Upon imbibition, the major DNA lesions are oxidative DNA damage, single-strand breaks (SSBs), and DSBs. Oxidative DNA damage is mostly removed by DNA glycosylases, in particular FPG and OGG1 [59]. SSBs are resolved through the nucleotide excision repair (NER) pathway [60], whilst DSBs are repaired by homologous recombination or non-homologous end-joining [54,58]. Detection of DNA damage leads to a pause in cell cycle progression, allowing time for repair at early imbibition stages [58,61]. UV-induced lesions, such as CPDs and (6-4)PPs, are removed mainly via DNA photolyase in photosynthetic tissues, or via the NER pathway [60]. Although generally less prevalent in dry seeds, CPDs and (6-4)PPs become relevant in the context of physical priming as these lesions may trigger DDR signalling. DDR responses are particularly evident in aged seeds, which display delayed germination, reflecting the longer time required to properly repair DNA damage [59]. In addition, repair of DNA DSBs by DNA ligases is fundamental for longevity of primed seeds [54].

The roles of DDR in traditional seed priming have been extensively discussed recently [8,53,58]. Traditional priming up-regulates transcript and protein levels of OGG1 during pre-germinative metabolism across several species [58,62–65], suggesting a higher response capacity or sensitized genome surveillance. Thus, enhancement of DDR during priming can contribute to improved germination. However, it remains unclear whether these processes are strictly imbibition-dependent, or whether similar processes can occur even in the dry state.

In addition to oxidative damage caused by ROS, physical agents may induce different DNA lesions depending on their mode of action: ionising radiation and NTP treatments might trigger physical perturbations in the form of DSBs, whilst UV radiation primarily generates CPDs and (6-4)PPs. However, information on DNA damage caused by physical priming and whether it is sufficient to trigger downstream DDR is limited. Whilst NTP treatment induced detectable genotoxic effects in pea seeds [66,67], genotoxic effects from NTP seem to differ depending on the medium where the treatment occurs [15]. Despite the increasing understanding of UV damage on plants [68], evidence for DNA damage caused by UV priming is particularly limited (e.g. [69]). In vegetative tissues, DDR is mediated by an up-regulation of CPD photolyases, leading to enhanced abiotic stress tolerance following UV exposure [60,68]. Moreover, (6-4)PPs are rapidly repaired around promotor and transcription start sites of genes including *AtICK1* [60], a cell cycle regulation gene, safeguarding overall genome integrity. In a similar manner, limited early induction of UV photoproducts across primed seed cells could up-regulate DDR, though direct evidence for this remains lacking. Interestingly, tardigrades (small invertebrates capable of withstanding extreme abiotic stress in a dehydrated state) prevent DNA lesion accumulation under high doses of UV-C and rely on rapid DNA repair post-hydration [70]. An intriguing possibility is that dry seeds also resist excessive genome damage and display augmented DDR upon imbibition following physical priming, leading to improved germination performance.

So far, it is not fully understood whether physical agents penetrate beyond the seed coat inducing genotoxic signals in deeper tissues, or whether superficial effects in the seed coat are communicated deeper into the seed. The seed coat has protective elements including a cuticle layer and phenolic compounds that shield internal tissues from exogenous stressors [71]. However, this barrier may not be absolute. Evidence from *Arabidopsis* leaf tissues indicate that UV-B radiation penetrates beyond epidermal layers, affecting genome stability in the germline [72]. If similar penetration occurs in dry seeds, UV radiation could directly induce photoproducts in the endosperm or embryo, activating DDR pathways. Furthermore, ROS species induced from NTP or ionising radiation may penetrate more deeply, causing oxidative damage or DSBs in internal tissues.

Based on this emerging evidence, we suggest that DDR is not merely a damage repair system, but that it is potentially a genotoxic signalling pathway able to integrate genome integrity information and enhance subsequent stress responses. This perspective would place DDR as a potential hallmark of a successful priming treatment [53]. Despite this, it is still unknown whether it is possible that DDR activation can occur in hydrated pockets, in which case priming is established during treatment, or whether DNA lesions remain stable until seeds are imbibed, when an enhanced DDR is triggered. Comparing the expression profiles of relevant genes may reveal the involvement of different DDR pathways in dry vs early imbibition stages and thereby suggest where DNA damage is initially sensed and how genotoxic signalling pathways are communicated.

## Transcriptional reprograming in seeds after priming

Seed priming induces widespread transcriptional reprograming during treatment and pre-germinative metabolism, with modifications persisting into early seedling development and stress exposure. In traditional priming, transcriptomic analyses reveal dynamic responses specific to priming agents and species [35,73–75]. As mediators of physical priming effects, ROS and DDR could impose both direct and indirect effects on transcriptional activity. Amongst limited evidence reported so far, transcriptional effects on ROS metabolism and reserve mobilization have emerged as outcomes of physical priming. NTP treatment of dry spinach seeds increased expression of the starch degrading enzyme, *pullulanase*, during germination [76], and improved the germination percentage of rice seeds affected by heat stress, also through an up-regulation of genes involved in starch metabolism [28]. Beyond these metabolic processes, physical priming also appears to target stress responsive genes. NTP-treated wheat had improved germination and seedling growth parameters under drought stress mediated by an up-regulation of drought responsive genes [33].

HSPs have been implicated in seed priming through protective functions [53,77], with roles in maintaining seed vigour and longevity [74,78]. However, evidence for their functional involvement in physical priming remains limited and inconsistent. NTP up-regulated HSP101 and HSP70 during early imbibition in maize [79], but no changes were detected in sunflower seeds following NTP or electromagnetic field treatments [80]. Notably, the WRKY transcription factor family is consistently implicated in physical priming. WRKY factors are associated with multiple responses, including salt, heat, and drought stress tolerance [81]. In hemp, 6 h post-imbibition NTP treatment induced persistent up-regulation of *WRKY1* and secondary metabolite biosynthesis genes, detectable in 30-day-old plants [43]. Similarly, rice plants from NTP-primed seeds exhibited *WRKY29* up-regulation, influencing stress responses and phytohormone production [82]. Lastly, NTP-primed *Andrographis paniculata* seeds exhibited greater germination and *WRKY33* was implicated in the regulation of abscisic acid and ethylene signalling [24]. The underlying basis for WRKY up-regulation upon priming remains unclear. There is evidence that overexpression of *OsWRKY89* enhanced UV-B tolerance and secondary metabolite accumulation [83], and multiple WRKY genes are induced by UV-B in *Arabidopsis* [84]. Additionally, nine WRKY transcription factors modulated flavonoid biosynthesis upon UV-B stress [85]. It is possible that UV-photons from NTP lead to UV-directed DDR and further ROS signalling, culminating in altered WRKY activity. Regardless of the upstream signal, WRKY genes are strong candidates for the coordination of the priming phenotype, at least for NTP.

A plausible basis for the maintenance of priming is through epigenetic modifications, enabling stress-responsive transcriptional states to persist beyond the germination stage. Epigenetic changes have been widely documented in traditional priming, and since DNA methylation patterns are influenced by endogenous stress responses, including ROS and DDR [54,61], long-term effects of physical priming could be mediated by DNA methylation [86]. For example, NTP priming of dry rice seeds induced DNA methylation modifications in the promoter regions of hormone and starch degradation genes [28], rescuing germination under heat stress. Ultrasonication priming of winter wheat seeds promoted early seedling growth that was linked to genome-wide DNA hypomethylation and significant transcriptional changes of hormonal and metabolic pathways [34]. Notably, hypomethylation increased DNA accessibility for the up-regulation of these key genes. Similarly, it was also demonstrated that *Arabidopsis* seeds primed with oxygen plasma irradiation undergo methylation modifications mediated by the down-regulation of two genes (*H1.2* and *RDM2*) related to chromatin structure and transcriptional repression [87]. The impact of chromatin remodelling upon signal onset during physical priming is still unclear, but DDR-mediated chromatin remodelling provides a mechanistic framework. UV-B exposure triggers chromatin remodelling essential for DNA repair in *Arabidopsis* and maize plants [88], and a similar effect has been described in mammalian cells where chromatin accessibility is essential for DDR [61].

Overall, available evidence indicates that physical priming elicits transcriptional reprogramming, particularly affecting hormonal and stress-responsive pathways. Additionally, epigenetic modifications may provide a molecular basis for the persistence of physical priming effects beyond germination.

## A roadmap to exploit physical priming

**Figure 3:**
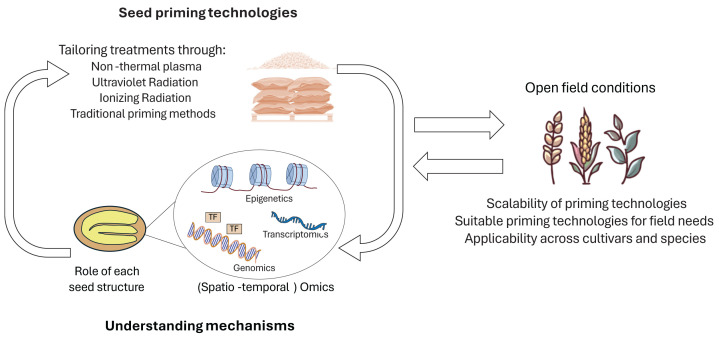
Schematic representation of the roadmap for exploiting physical seed priming technologies The framework depicts a cycle from understanding mechanisms across the seed to identify molecular markers of successful priming and optimising seed priming technologies to face specific needs. The most relevant step is the transition into open field conditions to determine the effectiveness of the technology.

Physical seed priming has grown substantially over recent years and can alter both germination and/or post-germination traits. It is clear that seed priming can be achieved independently of imbibition, representing a promising crop management tool against climate change. Despite this early achievement, two main areas require further development for a holistic understanding (Figure 3).

### Spatio-temporal control mechanisms

Although changes at the molecular level have been reported, such studies are limited. Whilst physical seed priming effects can persist beyond germination, it remains unclear whether they are maintained in later developmental stages and to what extent priming effects vary between different species or even cultivars. Future research must address relevant questions focused on the perception of physical stimuli and information storage. Identifying the roles played by each seed structure (seed coat, endosperm, and embryo) in the transmission and storage of the information is also important, yet might pose significant technical challenges. Evidence so far points to ROS and DDR as possible mechanisms underlying priming responses, but the relevant spatio-temporal organisation is poorly understood. Characterising this process will require the implementation of advanced omics analysis (see [89]) in addition to (non-) invasive imaging and single cell- or tissue-specific omics analyses. Fine scale dissection of the events associated with priming will provide an ideal framework for identifying seed priming hallmarks. Another layer of complexity is that such hallmarks should ideally be consistent across low- and high-quality seed genotypes of the same or different species.

### Large-scale (open field) conditions

While studies under standardised conditions are important, a transition into open field conditions is an essential requirement for capturing the effectiveness of the physical priming technology in real-world conditions. However, this transition is still in its early stages. Early greenhouse trials demonstrated significant priming benefits using NTP in crops such as tomato [32] and rapeseed [27]. Similarly, BioLumic—a New Zealand biotechnology company—reported yield increases in maize from UV-B primed seeds under field conditions [90]. Despite these early reports, systematic field trials remain limited.

As a next step, pushing its potential requires the development and implementation of cost-effective high-throughput seed treatment devices. Some authors considered the logistics of NTP production, and operation costs were far below the use of chemical fertilizer required for improved growth [91,92]. Nevertheless, alongside efficacy trials, cost-benefit assessments should be conducted to assess the needs, available seed enhancement methods, and opportunities for commercialization of physical priming.

## Summary

- Physical priming improves germination and plant performance.
- Potential mechanisms for perception of physical priming treatments include physiochemical changes to seed coats, ROS, and DNA damage.
- Improved understanding of physical priming will facilitate commercial exploitation.

## Open Access

This article has been published open access under our Subscribe to Open programme, made possible through the support of our subscribing institutions, learn more here: https://portlandpress.com/pages/open_access_options_and_prices#conditional.

## Abbreviations

(6-4)PP: pyrimidine-pyrimidone (6-4) photoproduct
CPD: cyclobutene pyrimidine dimer
DDR: DNA-damage response
DSB: double-strand break
HSP: heat shock proteins
NER: nucleotide excision repair
NTP: non-thermal plasma
RNS: reactive nitrogen species
ROS: reactive oxygen species
UV: ultraviolet
UVR8: UV RESISTANCE LOCUS 8

## References

[1] Hultgren A., Carleton T., Delgado M., Gergel D.R., Greenstone M., Houser T. et al. (2025) Impacts of climate change on global agriculture accounting for adaptation. Nature 642, 644–652 10.1038/s41586-025-09085-w 40533541 PMC12176627

[2] Rezaei E.E., Webber H., Asseng S., Boote K., Durand J.L., Ewert F. et al. (2023) Climate change impacts on crop yields. Nat. Rev. Earth Environ. 4, 831–846 10.1038/s43017-023-00491-0

[3] Kopittke P.M., Menzies N.W., Wang P., McKenna B.A. and Lombi E. (2019) Soil and the intensification of agriculture for global food security. Environ. Int. 132, 105078 10.1016/j.envint.2019.105078 31400601

[4] Finch-Savage W.E. and Bassel G.W. (2016) Seed vigour and crop establishment: extending performance beyond adaptation. J. Exp. Bot. 67, 567–591 10.1093/jxb/erv490 26585226

[5] Mordor Intelligence. Seed Market Size & Share Analysis—Growth Trends and Forecast (2026–2031). Available from: https://www.mordorintelligence.com/industry-reports/seeds-industry Accessed date: 04/12/2025

[6] Bewley J.D. (1997) Seed germination and dormancy. Plant Cell 9, 1055–1066 10.1105/tpc.9.7.1055 12237375 PMC156979

[7] Christou A., Agathokleous E. and Fotopoulos V. (2022) Safeguarding food security: hormesis-based plant priming to the rescue. Curr. Opin. Environ. Sci. Health 28, 100374 10.1016/j.coesh.2022.100374

[8] Pagano A., Macovei A. and Balestrazzi A. (2023) Molecular dynamics of seed priming at the crossroads between basic and applied research. Plant Cell Rep. 42, 657–688 10.1007/s00299-023-02988-w 36780009 PMC9924218

[9] Carrillo-Reche J., Vallejo-Marín M. and Quilliam R.S. (2018) Quantifying the potential of ‘on-farm’ seed priming to increase crop performance in developing countries. A meta-analysis. Agron. Sustain. Dev. 38, 64 10.1007/s13593-018-0536-0

[10] Fabrissin I., Sano N., Seo M. and North H.M. (2021) Ageing beautifully: can the benefits of seed priming be separated from a reduced lifespan trade-off? J. Exp. Bot. 72, 2312–2333 10.1093/jxb/erab004 33512455

[11] Araújo S.D.S., Paparella S., Dondi D., Bentivoglio A., Carbonera D. and Balestrazzi A. (2016) Physical methods for seed invigoration: advantages and challenges in seed technology. Front. Plant Sci. 7, 646 10.3389/fpls.2016.00646 27242847 PMC4863893

[12] Bera K., Dutta P. and Sadhukhan S. (2022) Seed priming with non-ionizing physical agents: plant responses and underlying physiological mechanisms. Plant Cell Rep. 41, 53–73 10.1007/s00299-021-02798-y 34654949

[13] Volkova P.Yu., Bondarenko E.V. and Kazakova E.A. (2022) Radiation hormesis in plants. Curr. Opin. Toxicol. 30, 100334 10.1016/j.cotox.2022.02.007

[14] Waskow A., Howling A. and Furno I. (2021) Mechanisms of plasma-seed treatments as a potential seed processing technology. Front. Phys. 9, 617345 10.3389/fphy.2021.617345

[15] Mildaziene V., Ivankov A., Sera B. and Baniulis D. (2022) Biochemical and physiological plant processes affected by seed treatment with non-thermal plasma. Plants 11, 856 10.3390/plants11070856 35406836 PMC9003542

[16] Zhou R., Zhou R., Wang P., Xian Y., Mai-Prochnow A., Lu X. et al. (2020) Plasma-activated water: generation, origin of reactive species and biological applications. J. Phys. D Appl. Phys. 53, 303001 10.1088/1361-6463/ab81cf

[17] Ji J., Wang X., Wang G., Zhang J., Song W., Wang R. et al. (2024) UV-B-priming combined with the soil application of MWCNT enhances rice growth performance under salt stress. J. Plant Growth Regul. 43, 3846–3861 10.1007/s00344-024-11367-y

[18] Thomas T.T.D. and Puthur J.T. (2020) UV-B priming enhances specific secondary metabolites in *Oryza sativa* (L.) empowering to encounter diverse abiotic stresses. Plant Growth Regul. 92, 169–180 10.1007/s10725-020-00628-x

[19] Fgaier S., Aarrouf J., Lopez-Lauri F., Lizzi Y., Poiroux F. and Urban L. (2023) Effect of high salinity and of priming of non-germinated seeds by UV-C light on photosynthesis of lettuce plants grown in a controlled soilless system. Front. Plant Sci. 14, 1198685 10.3389/fpls.2023.1198685 37469782 PMC10352585

[20] Thabet M., Abou-Zeid M.A., Safhi F.A., Alwutayd K.M. and Khalifa W. (2023) Ultraviolet-C irradiation of wheat grains induces seedling resistance to leaf rust and powdery mildew disease. Ital. J. Agron. 18, 2201 10.4081/ija.2023.2201

[21] Villegas D., Sepúlveda-Hernández C., Salamé M.J. and Poupin M.J. (2025) Enhancing growth and salinity stress tolerance in Arabidopsis with low-dose gamma radiation priming through a hormesis approach. Plant Stress 16, 100834 10.1016/j.stress.2025.100834

[22] Song K.E., Park C.Y., Hong S.H., Chung J.-I., Kim M.C. and Shim S.-I. (2022) Beneficial effects of gamma-irradiation of quinoa seeds on germination and growth. Radiat. Environ. Biophys. 61, 465–477 10.1007/s00411-022-00986-2 35833987

[23] Mumtaz S., Javed R., Rana J.N., Iqbal M. and Choi E.H. (2024) Pulsed high power microwave seeds priming modulates germination, growth, redox homeostasis, and hormonal shifts in barley for improved seedling growth: unleashing the molecular dynamics. Free Radic. Biol. Med. 222, 371–385 10.1016/j.freeradbiomed.2024.06.013 38901500

[24] Tong J., He R., Tang X., Li M. and Wan J. (2020) Transcriptomic analysis of seed germination improvement of *Andrographis paniculata* responding to air plasma treatment. PloS One 15, e0240939 10.1371/journal.pone.0240939 33091041 PMC7580921

[25] Pauzaite G., Malakauskiene A., Nauciene Z., Zukiene R., Filatova I., Lyushkevich V. et al. (2018) Changes in Norway spruce germination and growth induced by pre‐sowing seed treatment with cold plasma and electromagnetic field: short‐term versus long‐term effects. Plasma Process. Polym. 15, 1700068 10.1002/ppap.201700068

[26] Shahabi Z.M., Nasibi F. and Noori H. (2025) Cold plasma technology as a pre-treatment for seed priming enhances germination and reduces salinity stress in *Prosopis koelziana*. Sci. Rep. 15, 26250 10.1038/s41598-025-11637-z 40684025 PMC12276322

[27] Li L., Zhang L. and Dong Y. (2025) Seed priming with cold plasma mitigated the negative influence of drought stress on growth and yield of rapeseed (*Brassica napus* L.). Ind. Crops Prod. 228, 120899 10.1016/j.indcrop.2025.120899

[28] Suriyasak C., Hatanaka K., Tanaka H., Okumura T., Yamashita D., Attri P. et al. (2021) Alterations of DNA methylation caused by cold plasma treatment restore delayed germination of heat-stressed rice (*Oryza sativa* L.) seeds. ACS Agric. Sci. Technol. 1, 5–10 10.1021/acsagscitech.0c00070

[29] Adhikari B., Adhikari M., Ghimire B., Adhikari B.C., Park G. and Choi E.H. (2020) Cold plasma seed priming modulates growth, redox homeostasis and stress response by inducing reactive species in tomato (*Solanum lycopersicum*). Free Radic. Biol. Med. 156, 57–69 10.1016/j.freeradbiomed.2020.06.003 32561321

[30] Li K., Zhong C., Shi Q., Bi H. and Gong B. (2021) Cold plasma seed treatment improves chilling resistance of tomato plants through hydrogen peroxide and abscisic acid signaling pathway. Free Radic. Biol. Med. 172, 286–297 10.1016/j.freeradbiomed.2021.06.011 34139310

[31] Salami S., Ghorbani A., Rostami M., Mazandarani A. and Koolivand D. (2026) Sustainable cold plasma suppresses tomato brown rugose fruit virus in tomato seeds. Physiol. Mol. Plant Path. 142, 103123 10.1016/j.pmpp.2026.103123

[32] Jiang J., Lu Y., Li J., Li L., He X., Shao H. et al. (2014) Effect of seed treatment by cold plasma on the resistance of tomato to *Ralstonia solanacearum* (bacterial wilt). PloS One 9, e97753 10.1371/journal.pone.0097753 24840508 PMC4026385

[33] Guo Q., Wang Y., Zhang H., Qu G., Wang T., Sun Q. et al. (2017) Alleviation of adverse effects of drought stress on wheat seed germination using atmospheric dielectric barrier discharge plasma treatment. Sci. Rep. 7, 16680 10.1038/s41598-017-16944-8 29192193 PMC5709406

[34] Hidvégi N., Gulyás A. and Dobránszki J. (2022) Ultrasound, as a hypomethylating agent, remodels DNA methylation and alters mRNA transcription in winter wheat (*Triticum aestivum* L.) seedlings. Physiol. Plant. 174, e13777 10.1111/ppl.13777 36073119 PMC9826007

[35] Srivastava A.K., Suresh Kumar J. and Suprasanna P. (2021) Seed ‘primeomics’: plants memorize their germination under stress. Biol. Rev. 96, 1723–1743 10.1111/brv.12722 33961327

[36] Gómez-Ramírez A., López-Santos C., Cantos M., García J.L., Molina R., Cotrino J. et al. (2017) Surface chemistry and germination improvement of quinoa seeds subjected to plasma activation. Sci. Rep. 7, 5924 10.1038/s41598-017-06164-5 28725039 PMC5517418

[37] Stolárik T., Henselová M., Martinka M., Novák O., Zahoranová A. and Černák M. (2015) Effect of low-temperature plasma on the structure of seeds, growth and metabolism of endogenous phytohormones in pea (*Pisum sativum* L.). Plasma Chem. Plasma Process. 35, 659–676 10.1007/s11090-015-9627-8

[38] Pawlat J., Starek A., Sujak A., Kwiatkowski M., Terebun P. and Budzeń M. (2018) Effects of atmospheric pressure plasma generated in GlidArc reactor on *Lavatera thuringiaca* L. seeds’ germination. Plasma Process. Polym. 15, 1700064 10.1002/ppap.201700064 PMC5890984 29630623

[39] Bafoil M., Le Ru A., Merbahi N., Eichwald O., Dunand C. and Yousfi M. (2019) New insights of low-temperature plasma effects on germination of three genotypes of *Arabidopsis thaliana* seeds under osmotic and saline stresses. Sci. Rep. 9, 8649 10.1038/s41598-019-44927-4 31209339 PMC6572809

[40] Guajardo‐Flores D., Serna‐Guerrero D., Serna‐Saldívar S.O. and Jacobo‐Velázquez D.A. (2014) Effect of germination and UV‐C radiation on the accumulation of flavonoids and saponins in black bean seed coats. Cereal Chem. 91, 276–279 10.1094/CCHEM-08-13-0172-R

[41] August J., Bailly C. and Dufour T. (2024) Treatment of seeds by cold ambient air plasma: combining impedance measurements with water sorption modeling to understand the impact of seed hydration. J. Phys. D Appl. Phys. 57, 265203 10.1088/1361-6463/ad3838

[42] Kitazaki S., Sarinont T., Koga K., Hayashi N. and Shiratani M. (2014) Plasma induced long-term growth enhancement of *Raphanus sativus* L. using combinatorial atmospheric air dielectric barrier discharge plasmas. Curr. Appl. Phys. 14, S149–S153 10.1016/j.cap.2013.11.056

[43] Iranbakhsh A., Oraghi Ardebili Z., Molaei H., Oraghi Ardebili N. and Amini M. (2020) Cold plasma up-regulated expressions of WRKY1 transcription factor and genes involved in biosynthesis of cannabinoids in hemp (*Cannabis sativa* L.). Plasma Chem. Plasma Process. 40, 527–537 10.1007/s11090-020-10058-2

[44] Wang Y., Sun X., Peng J., Li F., Ali F. and Wang Z. (2025) Regulation of seed germination: ROS, epigenetic, and hormonal aspects. J. Adv. Res. 71, 107–125 10.1016/j.jare.2024.06.001 38838783 PMC12126707

[45] Wojtyla Ł., Lechowska K., Kubala S. and Garnczarska M. (2016) Different modes of hydrogen peroxide action during seed germination. Front. Plant Sci. 7, 66 10.3389/fpls.2016.00066 26870076 PMC4740362

[46] Grainge G., Nakabayashi K., Steinbrecher T., Kennedy S., Ren J., Iza F. et al. (2022) Molecular mechanisms of seed dormancy release by gas plasma-activated water technology. J. Exp. Bot. 73, 4065–4078 10.1093/jxb/erac150 35427417 PMC9232203

[47] Rahman M.M., Sajib S.A., Rahi M.S., Tahura S., Roy N.C., Parvez S. et al. (2018) Mechanisms and signaling associated with LPDBD plasma mediated growth improvement in wheat. Sci. Rep. 8, 10498 10.1038/s41598-018-28960-3 30002439 PMC6043519

[48] Cui D., Yin Y., Li H., Hu X., Zhuang J., Ma R. et al. (2021) Comparative transcriptome analysis of atmospheric pressure cold plasma enhanced early seedling growth in *Arabidopsis thaliana*. Plasma Sci. Technol. 23, 085502 10.1088/2058-6272/ac0686

[49] Leubner‐Metzger G. (2005) *β*‐1,3‐glucanase gene expression in low‐hydrated seeds as a mechanism for dormancy release during tobacco after‐ripening. Plant J. 41, 133–145 10.1111/j.1365-313X.2004.02284.x 15610356

[50] Zandalinas S.I. and Mittler R. (2018) ROS-induced ROS release in plant and animal cells. Free Radic. Biol. Med. 122, 21–27 10.1016/j.freeradbiomed.2017.11.028 29203327

[51] Jurdak R., Rodrigues G.D.A.G., Chaumont N., Schivre G., Bourbousse C., Barneche F. et al. (2022) Intracellular reactive oxygen species trafficking participates in seed dormancy alleviation in Arabidopsis seeds. New Phytol. 234, 850–866 10.1111/nph.18038 35175638

[52] Bailly C. (2019) The signalling role of ROS in the regulation of seed germination and dormancy. Biochem. J. 476, 3019–3032 10.1042/BCJ20190159 31657442

[53] Macovei A., Pagano A., Duenas C. Jr, Araujo S. and Balestrazzi A. (2025) Exploring the role of DNA damage response in seed priming to uncover key players for multi-stress tolerance. J. Exp. Bot. 77, 1953–1975 10.1093/jxb/eraf237 PMC13022554 40437782

[54] Waterworth W.M., Wang D., Dsilva L.S. and West C.E. (2025) DNA double strand break repair is important for the longevity of primed seeds. Plant Cell Environ. 48, 8469–8482 10.1111/pce.70142 40842195 PMC12586901

[55] Gohari G., Spanos A., Ioannou A., Efstathiou I., Panahirad S., Kolbert Z. et al. (2025) Seed priming approaches for climate-resilient agriculture. J. Exp. Bot. 77, 2013–2026 10.1093/jxb/eraf440 PMC13022540 41077714

[56] Kranner I., Minibayeva F.V., Beckett R.P. and Seal C.E. (2010) What is stress? Concepts, definitions and applications in seed science New Phytol. 188, 655–673 10.1111/j.1469-8137.2010.03461.x 20854396

[57] Herbst J., Li Q.-Q. and De Veylder L. (2024) Mechanistic insights into DNA damage recognition and checkpoint control in plants. Nat. Plants 10, 539–550 10.1038/s41477-024-01652-9 38503962

[58] Waterworth W., Balobaid A. and West C. (2024) Seed longevity and genome damage. Biosci. Rep. 44, BSR20230809 10.1042/BSR20230809 38324350 PMC11111285

[59] Waterworth W.M., Bray C.M. and West C.E. (2019) Seeds and the art of genome maintenance. Front. Plant Sci. 10, 706 10.3389/fpls.2019.00706 31214224 PMC6554324

[60] Kaya S., Erdogan D.E., Sancar A., Adebali O. and Oztas O. (2024) Global repair is the primary nucleotide excision repair subpathway for the removal of pyrimidine-pyrimidone (6-4) damage from the Arabidopsis genome. Sci. Rep. 14, 3308 10.1038/s41598-024-53472-8 38332020 PMC10853524

[61] Liu J., Liu L., He J., Xu Y. and Wang Y. (2021) Multi-omic analysis of altered transcriptome and epigenetic signatures in the UV-induced DNA damage response. DNA Repair (Amst.) 106, 103172 10.1016/j.dnarep.2021.103172 34298489

[62] Forti C., Shankar A., Singh A., Balestrazzi A., Prasad V. and Macovei A. (2020) Hydropriming and biopriming improve *Medicago truncatula* seed germination and upregulate DNA repair and antioxidant genes. Genes 11, 242 10.3390/genes11030242 32106615 PMC7140799

[63] Chen H., Chu P., Zhou Y., Li Y., Liu J., Chu P. et al. (2012) Overexpression of AtOGG1, a DNA glycosylase/AP lyase, enhances seed longevity and abiotic stress tolerance in *Arabidopsis*. J. Exp. Bot. 63, 4107–4121 10.1093/jxb/ers093 22473985

[64] Kiran K.R., Deepika V.B., Swathy P.S., Prasad K., Kabekkodu S.P., Murali T.S. et al. (2020) ROS-dependent DNA damage and repair during germination of NaCl primed seeds. J. Photochem. Photobiol. B: Bio. 213, 112050 10.1016/j.jphotobiol.2020.112050 33075649

[65] Ducatti K.R., Batista T.B., Hirai W.Y., Luccas D.A., Moreno L.D.A., Guimarães C.C. et al. (2022) Transcripts expressed during germination *sensu stricto* are associated with vigor in soybean seeds. Plants 11, 1310 10.3390/plants11101310 35631735 PMC9147077

[66] Kyzek S., Holubová Ľ., Medvecká V., Tomeková J., Gálová E. and Zahoranová A. (2019) Cold atmospheric pressure plasma can induce adaptive response in pea seeds. Plasma Chem. Plasma Process. 39, 475–486 10.1007/s11090-018-9951-x

[67] Tomeková J., Kyzek S., Medvecká V., Gálová E. and Zahoranová A. (2020) Influence of cold atmospheric pressure plasma on pea seeds: DNA damage of seedlings and optical diagnostics of plasma. Plasma Chem. Plasma Process. 40, 1571–1584 10.1007/s11090-020-10109-8

[68] Asik E., Kashif S.Z. and Oztas O. (2026) Plant tolerance mechanisms to DNA-damaging UV stress. J. Exp. Bot. 77, 27–55 10.1093/jxb/eraf272 PMC12707071 40581746

[69] Shapira Y., Bormashenko E. and Drori E. (2019) Pre-germination plasma treatment of seeds does not alter cotyledon DNA structure, nor phenotype and phenology of tomato and pepper plants. Biochem. Biophys. Res. Commun. 519, 512–517 10.1016/j.bbrc.2019.09.034 31530387

[70] Horikawa D.D., Cumbers J., Sakakibara I., Rogoff D., Leuko S., Harnoto R. et al. (2013) Analysis of DNA repair and protection in the tardigrade *Ramazzottius varieornatus* and *Hypsibius dujardini* after exposure to UVC radiation. PloS One 8, e64793 10.1371/journal.pone.0064793 23762256 PMC3675078

[71] Radchuk V. and Borisjuk L. (2014) Physical, metabolic and developmental functions of the seed coat. Front. Plant Sci. 5, 510 10.3389/fpls.2014.00510 25346737 PMC4193196

[72] Ries G., Heller W., Puchta H., Sandermann H., Seidlitz H.K. and Hohn B. (2000) Elevated UV-B radiation reduces genome stability in plants. Nature 406, 98–101 10.1038/35017595 10894550

[73] Gran P., Visscher T.W., Bai B., Nijveen H., Mahboubi A., Bakermans L.L. et al. (2025) Unravelling the dynamics of seed‐stored mRNAs during seed priming. New Phytol. 247, 2196–2209 10.1111/nph.70098 40152198 PMC12329163

[74] Barbosa Batista T., Javier Fernandez G., Alexandre Da Silva T., Maia J. and Amaral Da Silva E.A. (2020) Transcriptome analysis in osmo-primed tomato seeds with enhanced longevity by heat shock treatment. AoB Plants 12, plaa041 10.1093/aobpla/plaa041 32968476 PMC7494243

[75] Wang Y., Zhou E., Yao M., Xue D., Zhao N., Zhou Y. et al. (2023) PEG-6000 priming improves aged soybean seed vigor via carbon metabolism, ROS scavenging, hormone signaling, and lignin synthesis regulation. Agronomy 13, 3021 10.3390/agronomy13123021

[76] Ji S.H., Ki S.H., Kang M.H., Choi J.S., Park Y., Oh J. et al. (2018) Characterization of physical and biochemical changes in plasma treated spinach seed during germination. J. Phys. D Appl. Phys. 51, 145205 10.1088/1361-6463/aab2a2

[77] Leprince O., Pellizzaro A., Berriri S. and Buitink J. (2016) Late seed maturation: drying without dying. J. Exp. Bot. 68, 827–841 10.1093/jxb/erw363 28391329

[78] Kaur H., Petla B.P., Kamble N.U., Singh A., Rao V., Salvi P. et al. (2015) Differentially expressed seed aging responsive heat shock protein OsHSP18.2 implicates in seed vigor, longevity and improves germination and seedling establishment under abiotic stress. Front. Plant Sci. 6, 713 10.3389/fpls.2015.00713 26442027 PMC4568394

[79] Holubová Ľ., Švubová R., Slováková Ľ., Bokor B., Chobotová Kročková V., Renčko J. et al. (2021) Cold atmospheric pressure plasma treatment of maize grains—induction of growth, enzyme activities and heat shock proteins. Int. J. Mol. Sci. 22, 8509 10.3390/ijms22168509 34445215 PMC8395187

[80] Mildažienė V., Aleknavičiūtė V., Žūkienė R., Paužaitė G., Naučienė Z., Filatova I. et al. (2019) Treatment of common sunflower (*Helianthus annu*s L.) seeds with radio-frequency electromagnetic field and cold plasma induces changes in seed phytohormone balance, seedling development and leaf protein expression. Sci. Rep. 9, 6437 10.1038/s41598-019-42893-5 31015543 PMC6478675

[81] Chen L., Song Y., Li S., Zhang L., Zou C. and Yu D. (2012) The role of WRKY transcription factors in plant abiotic stresses. Biochim. Biophys. Acta Gene Regul. Mech. 1819, 120–128 10.1016/j.bbagrm.2011.09.002 21964328

[82] Bian J.-Y., Guo X.-Y., Lee D.H., Sun X.-R., Liu L.-S., Shao K. et al. (2024) Non-thermal plasma enhances rice seed germination, seedling development, and root growth under low-temperature stress. Appl. Biol. Chem. 67, 2 10.1186/s13765-023-00852-9

[83] Wang H., Hao J., Chen X., Hao Z., Wang X., Lou Y. et al. (2007) Overexpression of rice *WRKY89* enhances ultraviolet B tolerance and disease resistance in rice plants. Plant Mol. Biol. 65, 799–815 10.1007/s11103-007-9244-x 17960484

[84] Kilian J., Whitehead D., Horak J., Wanke D., Weinl S., Batistic O. et al. (2007) The AtGenExpress global stress expression data set: protocols, evaluation and model data analysis of UV‐B light, drought and cold stress responses. Plant J. 50, 347–363 10.1111/j.1365-313X.2007.03052.x 17376166

[85] Yu W., Zhou X., Meng J., Xu H. and Zhou X. (2025) WRKY transcription factors modulate the flavonoid pathway of *Rhododendron chrysanthum* Pall. under UV-B stress. Plants 14, 133 10.3390/plants14010133 39795393 PMC11723172

[86] Harris C.J., Amtmann A. and Ton J. (2023) Epigenetic processes in plant stress priming: open questions and new approaches. Curr. Opin. Plant Biol. 75, 102432 10.1016/j.pbi.2023.102432 37523900

[87] Nakano R., Tashiro K., Aijima R. and Hayashi N. (2016) Effect of oxygen plasma irradiation on gene expression in plant seeds induced by active oxygen species. Plasma Med. 6, 303–313 10.1615/PlasmaMed.2016019093

[88] Casati P., Campi M., Morrow D.J., Fernandes J.F. and Walbot V. (2011) Transcriptomic, proteomic and metabolomic analysis of UV-B signaling in maize. BMC Genomics 12, 321 10.1186/1471-2164-12-321 21679461 PMC3141669

[89] Auroux L. (2025) Advances in seed omics. J. Exp. Bot. 77, 2045–2058 10.1093/jxb/eraf294 PMC13022538 40580085

[90] BioLumic Limited. Available from: https://www.biolumic.com/turning-on-seeds Accessed date: 22/01/2026

[91] Gao X., Zhang A., Héroux P., Sand W., Sun Z., Zhan J. et al. (2019) Effect of dielectric barrier discharge cold plasma on pea seed growth. J. Agric. Food Chem. 67, 10813–10822 10.1021/acs.jafc.9b03099 31490069

[92] Khamsen N., Onwimol D., Teerakawanich N., Dechanupaprittha S., Kanokbannakorn W., Hongesombut K. et al. (2016) Rice (*Oryza sativa* L.) seed sterilization and germination enhancement via atmospheric hybrid nonthermal discharge plasma. ACS Appl. Mater. Interfaces 8, 19268–19275 10.1021/acsami.6b04555 27404121

